# Prospective evaluation of quality of life 54 months after high-dose intensity-modulated radiotherapy for localized prostate cancer

**DOI:** 10.1186/1748-717X-8-53

**Published:** 2013-03-06

**Authors:** Aurore Goineau, Virginie Marchand, Jérome Rigaud, Sylvain Bourdin, Emmanuel Rio, Loic Campion, Angélique Bonnaud-Antignac, Marc-André Mahé, Stéphane Supiot

**Affiliations:** 1 Integrated Center of Oncology René Gauducheau, Nantes-St Herblain, France; 2 Department of Urology, Hotel Dieu Hospital, University of Nantes, Nantes, France; 3 Department of Research in Psycho-oncology, Faculty of Medicine, University of Nantes, Nantes, France

**Keywords:** Prostate cancer, IMRT, Quality of life

## Abstract

**Objective:**

To determine late toxicity and quality of life (QoL) in patients with localized prostate cancer after high-dose intensity-modulated radiotherapy (IMRT).

**Patient and methods:**

This was a prospective study in patients with localized prostate adenocarcinoma who had been treated by IMRT (76 Gy) between February and November 2006. Physicians scored acute and late toxicity using the Common Terminology Criteria for Adverse Events (version 3.0). Patients completed cancer and prostate-specific QoL questionnaires (EORTC QLQ-C30 and QLQ-PR25) before IMRT (baseline) and at 2, 6, 18 and 54 months.

**Result:**

Data were available for 38 patients (median age, 73 years) (18% low risk; 60% intermediate risk; 32% high risk). The incidence of urinary and gastrointestinal toxicity was respectively: immediately post IMRT: 36.8% and 23.7% (grade 1), 5.3% and 5.3% (grade 2), 2.6% and 0% (grade 3); at 18 months: 23.7% and 10.3% (grade 1), 26.3% and 13.2% (grade 2), 0% and 2.6% (grade 3); at 54 months: 34.2% and 23.7% (grade 1), 5.3% and 15.8% (grade 2), 5.3% and 0% (grade 3). At 54 months, significant worsening was reported by patients for 11/19 QoL items but the worsening was clinically relevant (>10 points) for 7 items only: physical, role as well as social functioning, fatigue, pain, dyspnoea and constipation. There was no significant difference between 54-month and baseline QoL scores for global health, gastrointestinal symptoms, treatment-related symptoms and sexual function. However, there was significant - but clinically non-relevant (<10 points) - worsening of urinary symptom.

**Conclusion:**

High-dose IMRT to the prostate with accurate patient positioning did not induce any clinically relevant worsening in late urinary and gastrointestinal QoL at 54 months. Impaired physical and role functioning may be related to age and comorbidities.

## Introduction

In patients with localized prostate cancer and a good prognosis, the efficacy of external beam is similar to that of surgery or brachytherapy [1-3]. Adjuvant hormonal therapy is often offered to patients with an intermediate or poor prognosis [3]. However, over recent years, radiotherapy (RT) has seen major advances such as the introduction of 3D conformal RT (3D-CRT) and, more recently, intensity-modulated radiation therapy (IMRT) and image-guided RT (IGRT) [4,5].The higher radiation doses that can be delivered to the prostate by these new techniques, whilst sparing at-risk organs, has improved progression-free survival and reduced acute and late toxicities [6-13].

Quality of life (QoL) is a key criterion in the choice of treatment for early prostate cancer but is difficult to assess despite the availability of validated questionnaires [14-16]. Studies tend to be retrospective [17]. Long-term QoL after IMRT has received attention in a limited number of prospective studies [18,19] even though adverse effects are known to occur late (over 30 months) [20,21].

In 2006, we initiated a prospective study of the toxicity and QoL associated with high-dose IMRT for localized prostate cancer. Results for 18 months of follow-up have been published [22]. We now describe longer term results (54 months) to confirm good tolerance of this treatment after a long-term follow-up.

## Patients and methods

This prospective study was performed in the Radiotherapy Department of the “Integrated Center of Oncology René Gauducheau” and included all consecutive patients with localised prostate cancer who were eligible for IMRT without nodal irradiation between February and December 2006. Absence of lymph node invasion was established by laparoscopic lymphadenectomy in patients with a >10% risk of invasion according to Partin’s tables [23]. Absence of bone metastases was established by bone scintigraphy.

The 5-field IMRT technique delivering a dose of 76 Gy in 38 fractions to the prostate and delineation of organ-at-risk contour limits and constraints have been described in an earlier publication [22]. The percent organ-at risk volume receiving 65 Gy (V65) was derived from dose-volume histograms for bladder and rectal tissue. Patient positioning was checked daily [24].

Patients at intermediate risk of relapse according to D’Amico’s classification [1] received concurrent adjuvant hormonal therapy with an LHRH analogue for 3 to 6 months. Treatment was continued for 2 to 3 years in high-risk patients.

Physicians scored treatment toxicity (general, urinary, gastrointestinal (GI) and sexual) using the Common Terminology Criteria for Adverse Events (CTCAE) toxicity scale (version 3.0) immediately after IMRT (acute toxicity) and at subsequent visits (6, 18 and 54 months). Patients completed the core and prostate cancer-specific modules of the EORTC QoL questionnaires (QLQ-C30 version 3.0 and QLQ-PR25) at their pre-treatment and post-treatment visits (months 2, 6, 18 and 54). Items were combined into several scales (from 1 to 100) according to EORTC rules. For the global health and function scales, a high score signalled a better QoL; for the symptom scales, a high score was indicative of a poorer QoL.

Changes in toxicity and QoL over time were analyzed by the Wilcoxon signed-rank test. Correlations between toxicity and clinical or dosimetric variables were sought using the Mantel-Haenszel test for linear association (categorical variables) or the Spearman correlation coefficient (continuous variables). A difference from baseline was considered statistically significant if the p value was < 0.01 and clinically relevant if the difference was > 10 points, as per Osoba interpretation of EORTC criteria [25-27]. Factors that might predict QoL were tested by the Kruskal-Wallis test (categorical variables) or the Spearman correlation coefficient (continuous variables).

## Results

From February to November 2006, 55 consecutive patients with localized prostate cancer underwent IMRT. The characteristics of the 38 patients with data for analysis at 54 months are given in Table 1. The remaining 17 patients were broken down as follows: 5 deaths from causes other than prostate cancer, one case of Alzheimer’s disease and 3 cases of advanced cancer other than prostate cancer (rectum, lung, Vater’s ampulla) precluding completion of the questionnaires, 3 refusals to complete the questionnaire at 54 months, and 5 lost to follow-up.

**Table 1:** Patients’ characteristics

**Characteristics**	**n (%)**
**Age at diagnosis**	
≤ 65 ans	9 (24%)
65-80 ans	28 (74%)
≥ 80 ans	1 (2%)
**Cardiovascular history**	15 (39%)
**Classification risk at diagnosis**	
low risk	17 (45%)
Intermediate risk	11 (29%)
High risk	10 (26%)
**Cancer status at 54 months**	
Remission	29 (76%)
Biological relapse	9 (24%)
high risk	7 (18%)
intermediate risk	1 (2%)
low risk	1 (2%)

Median age at baseline was 73 years (range 54–80). Among the 55 patients, 25 (45.5%) had received hormonal therapy: 4 for 3 months, 11 for 6 months, and 10 for 2 to 3 years. The characteristics of the 17 loss to follow-up patients were very similar to those of the 38 analysable patients at 54 months in terms of median age (73), initial D’Amico’s classification (47% low risk, 24% intermediate and 29% high risk) and acute asthenia (59% mostly grade 1 at 41%), urinary toxicity (53% grade 1 and 35% grade 2) and bowel toxicity (24% grade 1 and 12% grade 2). Among the 38 analysable patients, 29 (76.3%) were in remission for prostate cancer and 9 (23.7%) in biochemical relapse whilst on hormonal therapy. Of these 9 patients, 7 (78%) were high-risk patients according to D’Amico’s classification.

Immediately after IMRT, 60.5% (15/38) patients reported asthenia (39.5% grade 1). Its incidence decreased to 26.3% (10/38) by 6 months and 7.9% (3/38) by 18 months, but rose to 36.8% (28.9% grade 1) by 54 months.

**Figure 1:**
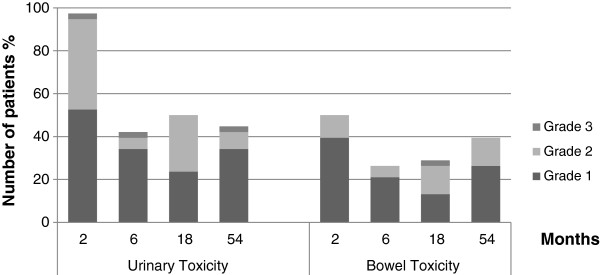
Physician assessment of urinary and bowel toxicity.

Physician-assessed urinary and GI toxicity over time is shown in Figure 1. During the last IMRT sessions, most patients suffered from urinary symptoms (52.6% grade 1; 42.1% grade 2), mainly from dysuria and increased urination frequency. Only one patient experienced grade 3 urinary toxicity and underwent placement of a temporary urinary catheter. Urinary toxicity resolved in 57.9% patients within 6 months. In the other patients, urinary toxicity was low grade (34.2% grade 1; 5.3% grade 2) except for one case (2.6%) of grade 3 toxicity. The incidence of urinary toxicity was highly similar at 6 and 18 months (42.1% and 50%, respectively) and remained stable until 54 months (44.7%) (34.2% grade 1; 7.9% grade 2) with one patient experiencing grade 3 toxicity. This patient underwent transurethral resection of the prostate (TURP) for dysuria with partial improvement of symptoms.

The reported incidence of GI toxicity immediately after IMRT was 50% (39.5% grade 1; 10.5% grade 2). The main symptoms were grade 1 diarrhoea (23.7%), flatulence (18% grade 1; 8% grade 2), grade 1 proctitis (15.8%) and haemorrhoids (n = 4 (10.5%) with 2 patients presenting slight rectal bleeding). The incidence decreased to 26.3% at 6 months, was 28.9% at 18 months, but rose to 39.5% (26.3% grade 1; 13.2% grade 2; no grade 3) at 54 months. At 54 months, diarrhoea was less frequent (10.6%) but had increased to grade 2 in 2 patients (5.3%). Flatulence was quite common (21.1% grade 1; 2.6% grade 2). Grade 1 rectitis and haemorrhoids affected 4 patients (10%) in each case.

Sexual function was not assessed in all patients at all time points. Only 12/38 patients (31.6%) were sexually active immediately after IMRT. Sexual dysfunction was recorded in 24/38 (63.2%) of patients at 18 months and 25/38 (65.8%) patients at 54 months (64% grade 1; 12% grade 2; 8% grade 3).

In an analysis of predictive factors for late toxicity at 54 months, acute toxicity was not predictive of general late toxicity (p = 0.52) or sexual dysfunction (p = 0.13). However, it was a predictive factor for both late urinary toxicity (p = 0.0094) and late GI toxicity (p = 0.0213). The overall rectal wall was not a predictive factor for late GI toxicity (p = 0.54) but the volume receiving 65 Gy (V65) or 60 Gy (V60) was (Table 2). Neither the overall nor V65 bladder wall volumes were predictive factors for late urinary toxicity (p = 0.93 and 0.99 respectively).

**Table 2:** Predictive factors of late toxicity

**Toxicity**	**Predictive factors**	**p**
General	Acute toxicity	NS
Urinary	Acute toxicity	0.01
	V65 bladder wall	NS
	V40 bladder wall	NS
	bladder wall volume	NS
Bowel	V65 rectal wall	0.04
	V60 rectal wall	0.03
	rectal wall volume	NS

**Figure 2:**
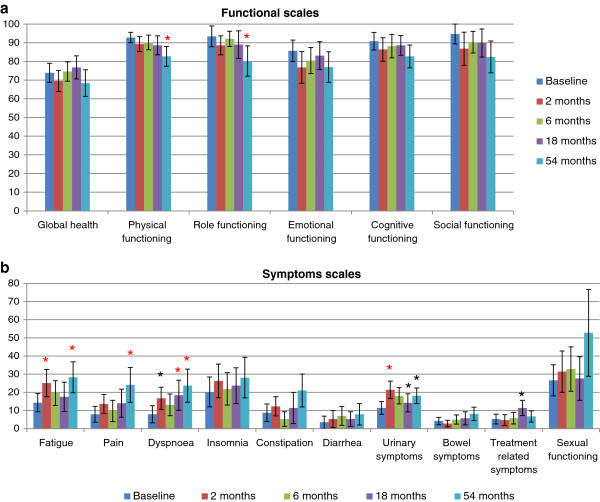
**Patient self-assessment of changes in quality of life over time (a) Functional scales, (b) Symptom scales.** *Significant difference with baseline (Wilcoxon signed rank test, p <0.05); * (in red) Statistically significant and clinically relevant difference (>10 points).

Results for patient-assessed QoL over time are illustrated in Figure 2 with data before treatment and at 2, 6, 18 and 54 months. There was no significant difference between time-points in global health, cognitive function, social or emotional functioning. Physical and role (i.e. work and play activities) functioning were maintained until 18 months but were decreased at 54 months (−10.1 points (p =0.0002) and-13.2 points (p = 0.003) respectively). Symptom scores at 54 months revealed increased fatigue (+14 points, p = 0.003), dyspnoea (+15.8 points, p = 0.002) and pain (+16.2 points, p = 0.0004). Urinary symptoms (dysuria, increased urination frequency, incontinence, urinary retention and haematuria) worsened at 2 months (+10 points, p = 0.0001) but then improved with time. The urinary symptom score was significantly higher at 54 months than immediately after IMRT but this difference was not clinically relevant as it was <10 points. No significant difference was recorded at any time in GI symptoms (diarrhoea, flatulence, rectitis, rectal bleeding, haemorrhoids) nor in sexual function.

In an analysis of predictive factors for QoL, risk category (D’Amico classification) was a predictive factor for global health (p = 0.026). Hormonal treatment was not (p = 0.34). Rectal wall volume (overall or V65) was not a predictive factor for GI symptoms. However, the V65 bladder wall volume was a predictive factor for urinary symptoms (p = 0.05). Overall bladder wall volume and risk category were not (p = 0.37 and 0.25, respectively).

## Discussion

In this 54-month prospective study of late toxicity and QoL in prostate cancer patients treated by IMRT, no significant difference of clinical relevance was observed between baseline and 54-month scores for several items of QoL (global health, cognitive, social and emotional functioning). However, in contrast to the 3-year data in the Lips et al. study, worsening physical and role functioning was observed at 54 months, which had not been present at 18 months [18,22]. The worsening we observed might be related to patient age. It may not necessarily be due to the presence or absence of hormonal therapy, as has been suggested [28].

We observed temporary impairment of QoL at 2 months mainly due to urinary disorders but urinary symptom scores improved thereafter [22]. The increase in score with respect to baseline at 54 months was not clinically relevant. According to the physician assessments, by 6 months, 57.9% of patients no longer experienced any urinary toxicity and the incidence remained relatively stable until 54 months (44.7%). According to the literature, the negative impact of urinary disorders on QoL has resolved by 12 months post RT [16,29], there is no difference in QoL due to urinary symptoms after IMRT without hormonal therapy compared to baseline, and urinary disorders impact less on QoL after IMRT than 3D-CRT [18,30].

At 54 months, patients reported significant and clinically relevant differences with respect to baseline in unspecific symptoms such as pain, dyspnoea and fatigue but no differences in prostate cancer-specific symptoms (urinary and GI disorders). The incidence of urinary and GI toxicity 54 months after 76 Gy IMRT in our study is similar to the 5-year incidence after 70 Gy 3D-CRT in the GETUG 06 trial [8]. Grade 2+ urinary toxicity occurred in 10.5% of our patients versus 10% (70 Gy) and 17.5% (80 Gy) in the GETUG 06 trial; Grade 2+ GI toxicity occurred in 13.2% of our patients versus 14% (70 Gy) and 19.5% (80 Gy) patients in the GETUG 06 trial [8]. Dose escalation without enhancing toxicity is thus possible if IMRT is used instead of 3D-CRT. The results of several other studies support this conclusion: a 16% incidence of urinary toxicity and 3% incidence of GI toxicity (grade 2+) 10 years after 81 Gy IMRT, a 9% incidence of grade 2 urinary toxicity and a 5% incidence of grade 2 GI toxicity 3 years after 76 Gy IMRT, and no difference in urinary or GI toxicity between 78 Gy IMRT and 70 Gy 3D-CRT [31-33].

There were no significant and clinically relevant differences in sexual function over time, in contrast to the observations of Lips et al., but our patient numbers were too small for the assessment to be meaningful [18,34]. Respondent numbers were lowest immediately after IMRT (n = 12) and at 6 months (n = 12) (compared to n = 24 and 25 at 18 and 54 months, respectively), suggesting that recovery of sexual activity occurs only some time after treatment. Assessment of sexual function should therefore probably not be restricted to assessment of just erectile dysfunction, the commonly used objective criterion, but should also take psychological factors into consideration. In addition, assessment can be beleaguered by the fact that a decrease in libido is sometimes still a taboo subject that the practitioner may have difficulty in bringing up with some patients [35].

Like other teams, we were able to identify few factors associated with late urinary and GI toxicity and QoL, probably because we had little data available for analysis at 54 months [36]. The only predictive factor in our study was acute toxicity. In particular, hormonal therapy was not an adverse predictive factor for QoL in our study, contrary to recent findings [37]. In our initial analysis at 18 m, ADT was associated with a worsened quality of life, especially dyspnea, insomnia, treatment related symptoms and sexual functioning. At 54 m, only 9 patients out of 36 were still receiving ADT. This small proportion of patients probably explains why we were not able to identify an adverse QoL impact from ADT. Furthermore, Pederson et al. found that age was associated with GI toxicity and whole-pelvic IMRT with genito-urinary toxicity at 41 months, and that rectal but not bladder dose constraints were associated with toxicity [32]. However, such constraints, even though they are crucial in limiting acute toxicity, are difficult to establish because bladder and rectal filling varies between sessions [38].

The strengths of our QoL study are its prospective design and long (4½ years) follow-up. A key methodological aspect is the use of validated self-administered patient questionnaires. However, an important shortcoming is the small number of patients who completed the 54-month questionnaire owing to the deaths (unrelated to prostate cancer) or serious illnesses encountered in this aged population. There was no control arm but the prospective design enabled comparisons with baseline.

## Conclusion

In conclusion, the high precision of IMRT in the treatment of localized prostate cancer enables dose escalation without engendering unacceptable toxicity [39]. There was no impairment of urinary, GI and sexual function nor of overall QoL at 54 months post IMRT. Decreased physical and role functioning were observed but may be related to ageing and comorbidities.

## Consent

Written informed consent was obtained from the patient for publication of this report and any accompanying images.

Presented at the 2012 ESTRO Annual Congress, Barcelona

## Competing interest

The authors declare that they have no competing interests.

## Authors’ contribution

AG, VM, JR, SB, LC, SS designed the research, AG, VM, ER, MAM and SS, performed the research, AG, VM, LC, ABA and SS analyzed the data, AG and SS wrote the paper. All authors read and approved the final manuscript.
